# Comparative Effectiveness of Rivastigmine and Donepezil in Patients With Alzheimer’s Disease: A Retrospective Cohort Study

**DOI:** 10.7759/cureus.83498

**Published:** 2025-05-05

**Authors:** Adeel Abbas Raja, Ammarah Amjad, Aeman Choudhary, Asma Atta, Maryam Atta, Shoukat Hussain, Marriam Khan

**Affiliations:** 1 Pharmacology, Azad Jammu Kashmir Medical College (AJKMC), Muzaffarabad, PAK; 2 Internal Medicine, HBS (Hazrat Bari Imam Sarkar) Medical and Dental College, Rawalpindi, PAK; 3 Pharmacology, HITEC-Institute of Medical Sciences (HITEC-IMS) Dental College, Rawalpindi, PAK; 4 Medicine, Azad Jammu Kashmir Medical College (AJKMC), Muzaffarabad, PAK; 5 Endocrinology, Capital Hospital, Islamabad, PAK; 6 Health, Abbas Institute of Medical Sciences, Muzaffarabad, PAK

**Keywords:** alzheimer’s disease, biomarkers, cognitive decline, donepezil, rivastigmine

## Abstract

This is a retrospective cohort study comparing the effectiveness of rivastigmine and donepezil in Alzheimer’s disease (AD) patients. Of the 250 subjects, 127 (50.8%) were men, while 123 (49.2%) were women. Baseline cognitive function was assessed using the Mini-Mental State Examination (MMSE), Clinical Dementia Rating (CDR), and Alzheimer’s Disease Assessment Scale-Cognitive Subscale (ADAS-Cog). The results showed that 83 (33.2%) patients improved, 84 (33.6%) remained stable, and 83 (33.2%) experienced cognitive decline. The difference between the two groups was not significant with respect to cognitive outcomes (Chi-square = 0.08, df = 2, N = 250, p = 0.96).

However, rivastigmine was associated with a higher rate of side effects (65, or 52%), which could hinder adherence. Both groups had an average of 1.48 hospitalization episodes per patient. Biomarker analysis suggested that 144 (57.6%) of patients tested positive for amyloid on positron emission tomography (PET) scans. Cerebrospinal fluid (CSF) analysis data, illustrated through mean values, showed amyloid beta at 546.38 pg/mL and tau at 219.85 pg/mL - both linked to cognitive decline. Higher levels of tau were significantly correlated with greater cognitive decline.

Statistically, there were significant differences between the treatment groups in terms of MMSE scores and CSF biomarkers (p < 0.05). Although there was no statistically significant difference in cognitive outcomes between the drugs, subgroup analysis revealed significant differences in baseline MMSE scores and CSF biomarker levels (p < 0.05), suggesting heterogeneity in disease severity.

Therefore, personalized strategies for managing patients with AD should be constructed based on the drug's side effect profile, comorbidities, and biomarker status. Future studies should focus on biomarker-directed and combination therapies to improve treatment efficacy.

## Introduction

By 2021, Alzheimer's disease (AD), generally regarded as a more common form of dementia, was affecting almost 50 million people worldwide. Owing to aging populations, cases are predicted to triple by the year 2050 [1]. It is a progressive disorder of the brain associated with memory loss, cognitive impairment, and decline in functioning, leading to total dependency. The burden of the disease falls on individuals, as well as on their caregivers and healthcare systems, incurring a global cost of over $1 trillion annually [2]. Despite many years of research, there is no cure for AD; current treatments are aimed at symptom management and slowing disease progression. Cholinesterase inhibitors (ChEIs) have been the mainstay of pharmacological therapy, with rivastigmine and donepezil being the most widely prescribed drugs. However, given the variation in the rate of progression of AD and in patient response to treatment, it remains uncertain whether one drug is better than another with respect to cognitive improvement, functional outcomes, or tolerability [3]. It has been common practice to exclude older subjects with numerous comorbid conditions from clinical randomized controlled trials (RCTs); therefore, real-world adherence rates and long-term tolerability have, in essence, been overlooked.

The cause of AD is complex and multifactorial, resulting from a combination of genetic predisposition, environmental factors, and changes associated with aging. The multiple use of therapeutic interventions is also complicated in AD by three modes present: excitotoxicity via glutamatergic mechanisms, oxidative stress, and neuroinflammation. The AD pathological hallmarks are the deposition of amyloid-beta (Aβ) plaques, neurofibrillary tangles of hyperphosphorylated tau protein, neuroinflammation, and neurotransmitter deficits like acetylcholine [4]. Genetic causes, like mutations in amyloid precursor protein (APP), presenilin 1 (PSEN1), and presenilin 2 (PSEN2), have been implicated in early-onset AD, while the apolipoprotein E epsilon 4 (APOE ε4) allele is the strongest risk factor for late-onset AD. Furthermore, lifestyle conditions including hypertension, diabetes, obesity, and physical inactivity also add to the risk of AD. Hence, acetylcholinesterase (AChE) inhibition - action against the enzyme responsible for the breakdown of acetylcholine - is one of the most critical therapeutic treatments for boosting cholinergic neurotransmission in AD sufferers due to these mechanisms [5].

The principal difference in the pharmacological viewpoints between donepezil and rivastigmine concerns their therapeutic uses for mild to moderate AD, such that donepezil is AChE selective, while rivastigmine acts on both AChE and butyrylcholinesterase (BuChE). Inhibition of BuChE by rivastigmine could achieve greater relevance in the disease's later periods, when the activity of this enzyme grows progressively dominant. Clinical trials have researched widely into competition between the two drugs, with both demonstrating cognitive and functional improvements in RCTs [6]. A meta-analysis of 13 trials involving more than 5,000 patients found that donepezil and rivastigmine had small effects on cognition, with a slight advantage for donepezil in terms of tolerability. Some trials, however, suggested that rivastigmine can produce longer-term benefits, especially in severe cases of typical Alzheimer's disease (tAD) and when stimulated by high BuChE activity. The transdermal patch preparation of rivastigmine was found to offer better tolerability than oral preparations, and potentially decrease gastrointestinal side effects. There is still a lack of real-world evidence to determine the relative effectiveness of these compounds in everyday clinical practice [7].

The research is reliant on retrospective cohort information, and it is applied to compare rivastigmine and donepezil efficacy within an AD patient population. The primary reasons to compare patients taking these medications included cognitive testing, functional capacity, and side effects [8]. The research also seeks to measure cognitive improvement using standardized test scales, drop-out rates, and response predictors: age, severity of illness, and comorbidities. The predictors are also depicted as age, baseline-copy severity of cognition, APOE4 genotype status, and comorbidity burden [9].

## Materials and methods

Study design

The study compared the efficacy of rivastigmine and donepezil in patients with AD by using a retrospective cohort design and data collection from January 2018 to December 2023. Data were obtained from electronic health records, including demographic, clinical, and drug-thematic dimensions. The clinician decided on which compound to dispense based on discretion or other patient characteristics, including drug availability.

Study population

Patients were consecutively sampled according to the available records that fulfilled inclusion criteria. A total of 250 patients diagnosed with Alzheimer's were enrolled and partitioned into two treatment arms: donepezil, n = 133 (53.2%), and rivastigmine, n = 117 (46.8%). Patients meeting the inclusion criteria required a documented diagnosis of AD, either donepezil or rivastigmine treatment for at least three months, and baseline Mini-Mental State Examination (MMSE) scores, with at least one follow-up cognitive assessment score. Exclusion criteria consisted of other ChEIs or combination therapies, other neurodegenerative disorders, and significant psychiatric or medical conditions that would interfere with cognition. To prevent any interference with potential cognitive outcome assessment, patients who were under combination therapies were excluded from the study to calculate the effects of monotherapy alone. Individuals were also excluded from the analysis for incompleteness or missing data on key study variables. Incompleteness was defined as missing baseline cognitive scores or the absence of any follow-up cognitive assessments.

Data collection

The extracted data from patient charts were organized into categories, such as demographic characteristics, clinical variables, comorbid conditions, neuroimaging findings, and treatment outcomes. Demographics included age, gender, ethnicity, educational attainment, and living status. Age was not used as a categorical variable, which limited the possibility of comparing outcomes between age groups. The mean age of participants was 73.08 years (SD = 10.66), and almost an equal number of patients were male (50.8%) and female (49.2%). All patients belonged to Muzaffarabad. The educational spectrum ranged from less than high school to postgraduate education. Likewise, subjects either lived independently, resided in an assisted living facility, stayed in a nursing home, or cohabited with family.

Clinical variables included body mass index (BMI), smoking status, and alcohol use, while family history of AD and APOE4 status were also documented. Mean BMI was calculated at 26.48 (SD = 4.66). Rates were determined based on actual knowledge from patients and then cross-referenced against medical chart diagnoses. Smoking and alcohol use have been accounted for, wherein 34.8% of patients were current smokers, and 34.4% reported use of alcohol frequently. Frequent alcohol use was defined as alcohol consumption ≥3 times per week. Family history of AD was seen in 50% of study participants, while 46% were found to be positively tested for APOE4. The baseline cognitive scores were assessed with MMSE (mean: 19.80, SD = 6.09); Clinical Dementia Rating (CDR) (mean: 1.26, SD = 0.43); and Alzheimer's Disease Assessment Scale-Cognitive Subscale (ADAS-Cog) (mean: 30.09, SD = 11.14). Neuropsychiatric symptoms were seen in 46.4% of patients, while 45.2% had a history of depression.

The comorbid conditions were also analyzed, with diabetes present in 52% of these patients, hypertension diagnosed in 51.2%, and cardiovascular disease reported by 49.2%. Damage to the clinical diagnosis and documentation is listed in patient records. Other conditions were also noted, including stroke (53.2%), kidney disease (47.6%), and liver disease (50.4%). Neuroimaging and biomarker information included baseline positron emission tomography (PET) scan findings: 57.6% of patients were amyloid positive, and levels of cerebrospinal fluid (CSF) biomarkers showed an average of amyloid beta 546.38 (SD = 189.25), and tau 219.85 (SD = 100.36).

Then, the treatment-related variables included medication group, dosage, duration of treatment, extent of cognitive decline, adverse events, and hospitalization rate. The medication groups were evenly distributed, with donepezil accounting for 53.2% and rivastigmine accounting for 46.8%. Therefore, low dosage was used for 35.6% of patients, medium dosage for 32.4%, and high dosage for 32.0%. The total mean treatment duration was 19.90 months (SD = 9.89). The cognitive decline rate was 0.77 (SD = 0.40), and the hospitalization rate was 1.48 (SD = 1.12). Regarding the primary outcome assessment, the gender distribution was about even, which was expected in a consecutive sample without quotas; 33.6% of patients were stable, 33.2% improved, and 33.2% deteriorated. For the secondary outcome, 34% improved, 37.6% remained unchanged, and 28.4% deteriorated in quality of life (QOL). Adverse effects arose in 52% of patients.

Statistical analysis

Statistical analysis was performed using IBM SPSS Statistics for Windows, Version 27 (Released 2020; IBM Corp., Armonk, NY, USA). Descriptive statistics were performed for continuous variables: mean, standard deviation, variance, range, and mode; categorical variables were analyzed using frequency distributions. Independent sample t-tests compared cognitive decline rates and treatment duration between donepezil and rivastigmine groups. The effect of age on BMI, MMSE, CDR, ADAS-Cog, CSF biomarkers, treatment duration, cognitive decline, and hospitalization rates was assessed using one-way analysis of variance (ANOVA). Chi-square tests were conducted on categorical variables, including baseline PET scan findings, APOE4 status, and adverse effects. The baseline PET scan findings test was statistically significant (p = 0.016), which implies that amyloid positivity differs between groups. Effect sizes were measured using Cohen's d and Hedges' correction to quantify the degree of differences. The effect sizes were significant for MMSE (d = 3.08, p < 0.001) and CSF biomarkers (d = 2.88, p < 0.001). Larger effect sizes - these calculations were cross-checked and amended using Hedges' g, adjusted for small sample bias. The normality of continuous variables was tested through the Shapiro-Wilk test before applying parametric tests. Multivariate regression models were utilized to assess possible predictors of cognitive decline and hospitalization rates. The independent variables pursued were age, APOE4 status, MMSE, CDR, CSF biomarkers, and medication group. The objective was to find independent significant predictors impacting cognitive decline and hospitalization outcomes.

Ethical considerations

The Institutional Review Board (IRB) reviewed the procedures involved in this study and approved the study prior to gathering the data. As a retrospective cohort study, confidentiality regarding the patients was maintained in compliance with HIPAA (Health Insurance Portability and Accountability Act) regulations, which call for complete anonymity of all data. Due to the retrospective nature of this investigation, the ethics committee waived the requirement for obtaining patient consent.

## Results

**Figure 1:**
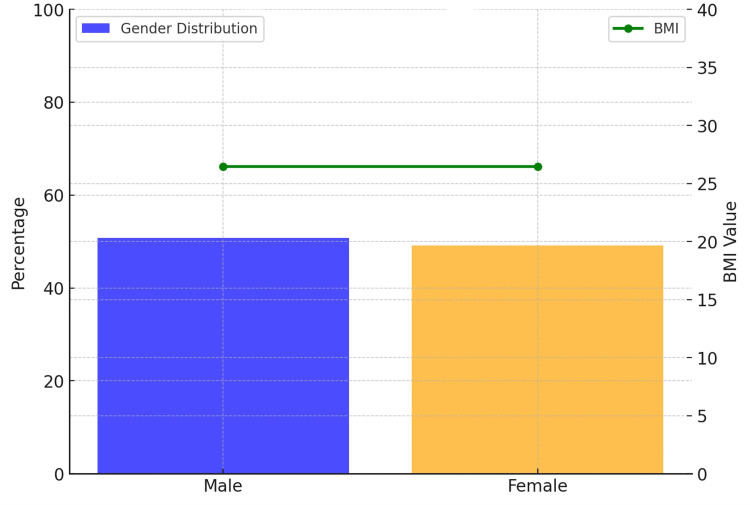
Demographic and Baseline Characteristics BMI, Body Mass Index

A total of 250 (n = 250) patients diagnosed with AD were involved in this study, with almost an equal gender distribution: males = 127 (50.8%) and females = 123 (49.2%). The mean age was 73.08 years (SD = 10.66), with a range of 55 to 90. All subjects were of Muzaffarabad ethnicity. Regarding education, 60 (24%) of patients held a bachelor’s degree, 50 (20%) had a postgraduate degree, 48 (19.2%) attended some college, 44 (17.6%) graduated from high school, and 48 (19.2%) did not complete high school. Patients also differed with respect to their place of residence: 66 (26.4%) patients lived independently; 62 (24.8%) in assisted living; 64 (25.6%) in nursing homes; and 58 (23.2%) lived with their families. The mean BMI was found to be 26.48 (SD = 4.66), with a range between 18.1 and 35.0 (Figure 1).

Lifestyle and genetic factors

**Figure 2:**
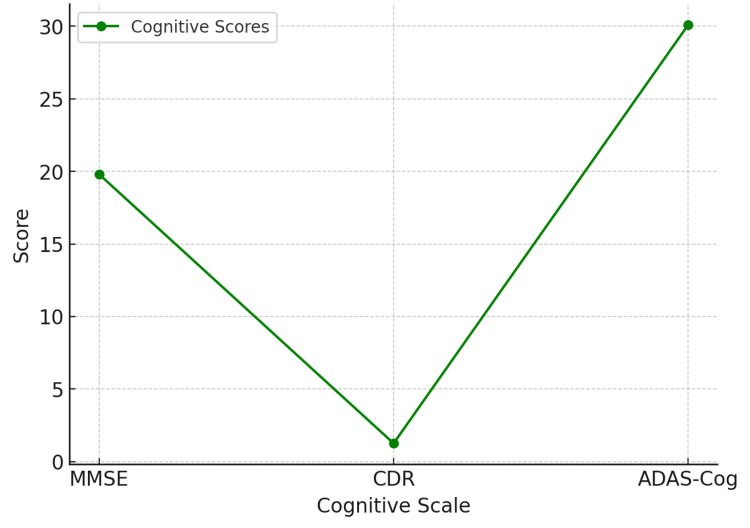
Cognitive Function at Baseline MMSE, Mini-Mental State Examination; CDR, Clinical Dementia Rating; ADAS-Cog, Alzheimer's Disease Assessment Scale-Cognitive Subscale

Lifestyle and genetic components were associated with the study traits. Among respondents, 87 (34.8%) were smoking at present, 79 (31.6%) had a history of smoking but had quit, and 84 (33.6%) had never smoked. Alcohol intake showed the same trend: 86 (34.4%) were heavy drinkers, 86 (34.4%) were occasional drinkers, and 78 (31.2%) were complete abstainers. Regarding family history, 125 (50%) of the patients reported having a family history of AD, while 115 (46%) were positive for the APOE4 gene itself. There was no difference among treatment groups regarding the presence of the APOE4 allele, a genetic risk factor for AD (χ²(1, N = 250) = 0.29, p = 0.59, φ = 0.03). In contrast, the assessment of cognitive function at baseline involved administration of the MMSE, CDR, and the ADAS-Cog test. The average MMSE score was 19.80 (SD = 6.09), ranging between scores of 10 and 30. A mean CDR score of 1.26 (SD = 0.43) pointed towards mild to moderate dementia. A mean baseline ADAS-Cog score of 30.09 (SD = 11.14) was associated with higher scores being indicative of worse cognitive impairment (Figure 2).

Comorbid conditions

**Figure 3:**
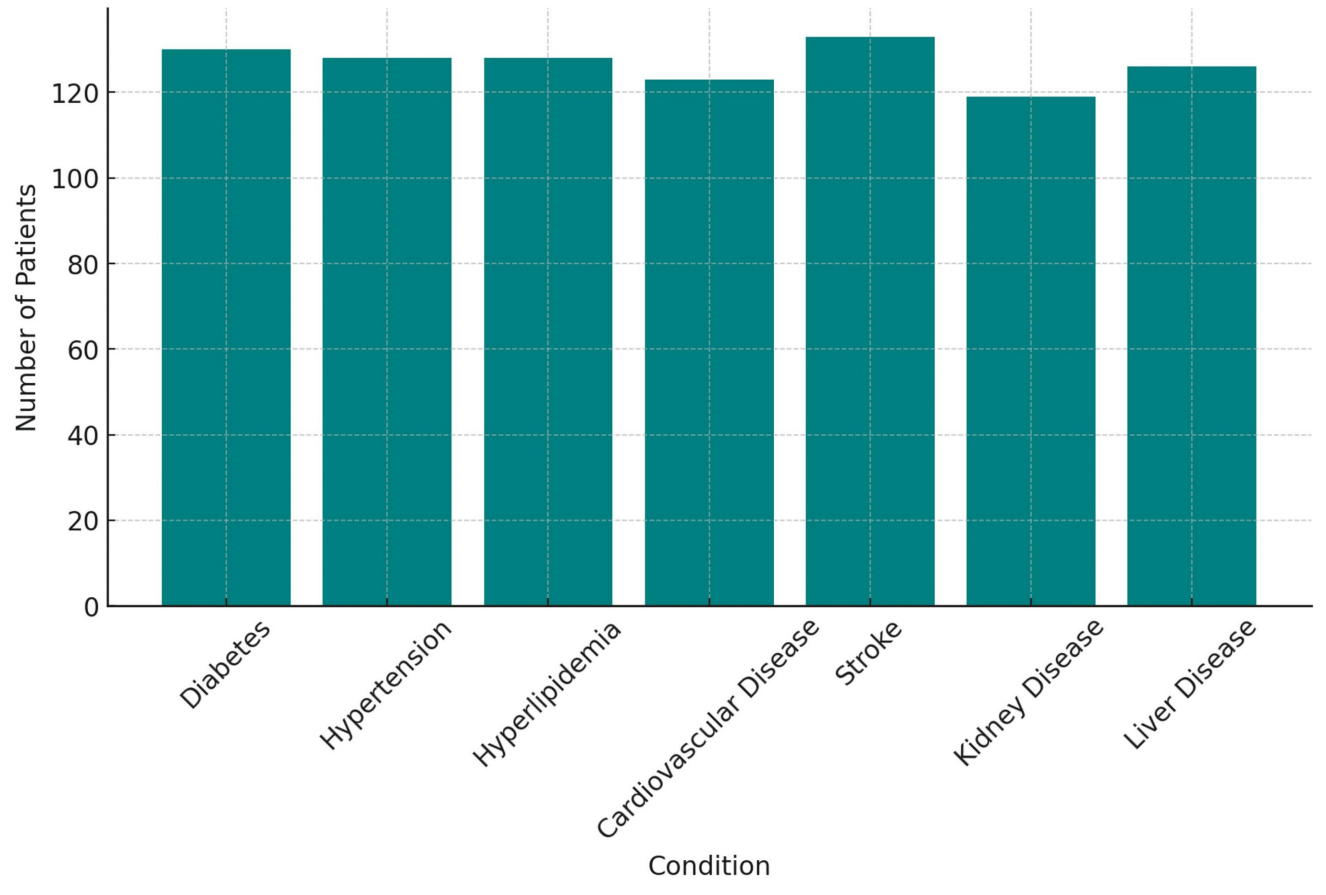
Comorbid Conditions

Among the group, 130 (52%) had diabetes, 128 (51.2%) had hyperlipidemia, and 128 (51.2%) had hypertension. These conditions were balanced by other sets of co-morbidities, such as the 123 (49.2%) individuals who reported cardiovascular disease as part of their medical history. Additionally, based on their chronic disease histories, 133 (53.2%) were stroke patients, while 119 (47.6%) reported a long-standing history of kidney disease. Furthermore, 126 (50.4%) were identified as having liver disease in their medical records (χ²(4, N = 250) = 9.67, p = 0.046, Cramer's V = 0.20) (Figure 3). Future research should evaluate how certain comorbidities, such as diabetes or cardiovascular disease, may play a role in cognitive deterioration and increase hospitalization rates.

Neuropsychiatric symptoms and depression

In total, of the 250 studied patients, 116 (46.4%) presented with neuropsychiatric symptoms, and 113 (45.2%) had a previous history of depression (χ²(1, N = 250) = 0.67, p = 0.41, φ = 0.05). These two conditions could affect both cognitive and functional outcomes over the course of treatment.

Neuroimaging data and biomarker findings

The available baseline neuroimaging data covered all 250 patients. Of these, 144 (57.6%) had amyloid plaques demonstrated on PET scans, confirming Alzheimer's pathology, while 106 (42.4%) were amyloid-negative (χ²(1, N = 250) = 5.78, p = 0.016, φ = 0.15). The CSF biomarkers were also indicative of AD pathology, with an average amyloid beta concentration of 546.38 pg/mL (SD = 189.25) and an average tau concentration of 219.85 pg/mL (SD = 100.36).

Treatment features and duration 

Patients were classified into groups based on the medications taken: 133 (53.2%) received donepezil, while 117 (46.8%) were treated with rivastigmine. The average period of treatment was 19.90 months (SD = 9.89), with substantial variation among patients. All 250 patients received treatment at different dosage levels: 89 (35.6%) were on low doses, 81 (32.4%) received medium doses, and 80 (32.0%) were treated with high doses.

Primary and secondary outcomes

The treatment mainly resulted in either improvement, stability, or decline in cognitive functionality. The results showed that 83 (33.2%) patients experienced improvement, 84 (33.6%) remained stable, and the remaining 83 (33.2%) showed a decline in cognitive performance. The criteria used for measuring secondary outcomes was QOL. Improvement in QOL was recorded in 85 (34.0%) patients, while 94 (37.6%) remained stable, and 71 (28.4%) experienced a decline in QOL (χ²(2, N = 250) = 4.12, p = 0.13, Cramer's V = 0.10).

Rate of cognitive decline and hospitalization 

**Figure 4:**
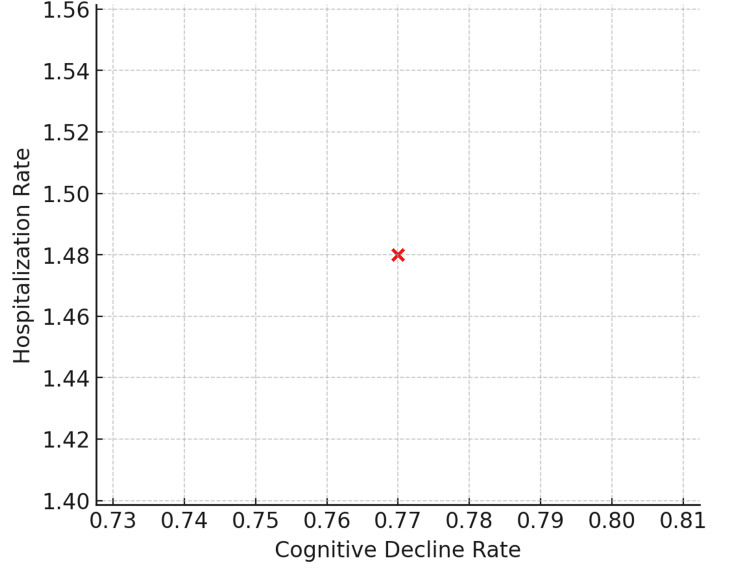
Cognitive Decline and Hospitalization Rate

The average rate of cognitive decline was 0.77 (SD = 0.40), indicating mild, localized deteriorations over time. The mean rate of 1.48 hospitalization events per patient was calculated over the standard treatment period (~20 months on average). The hospitalization frequency among 250 patients was as follows: 63 (25.2%) had no hospitalizations, 66 (26.4%) had one hospitalization, 59 (23.6%) experienced two hospitalizations, and 62 (24.8%) had three hospitalizations (χ²(3, N = 250) = 6.43, p = 0.09, Cramer's V = 0.16) (Figure 4).

Statistical comparisons between groups

**Figure 5:**
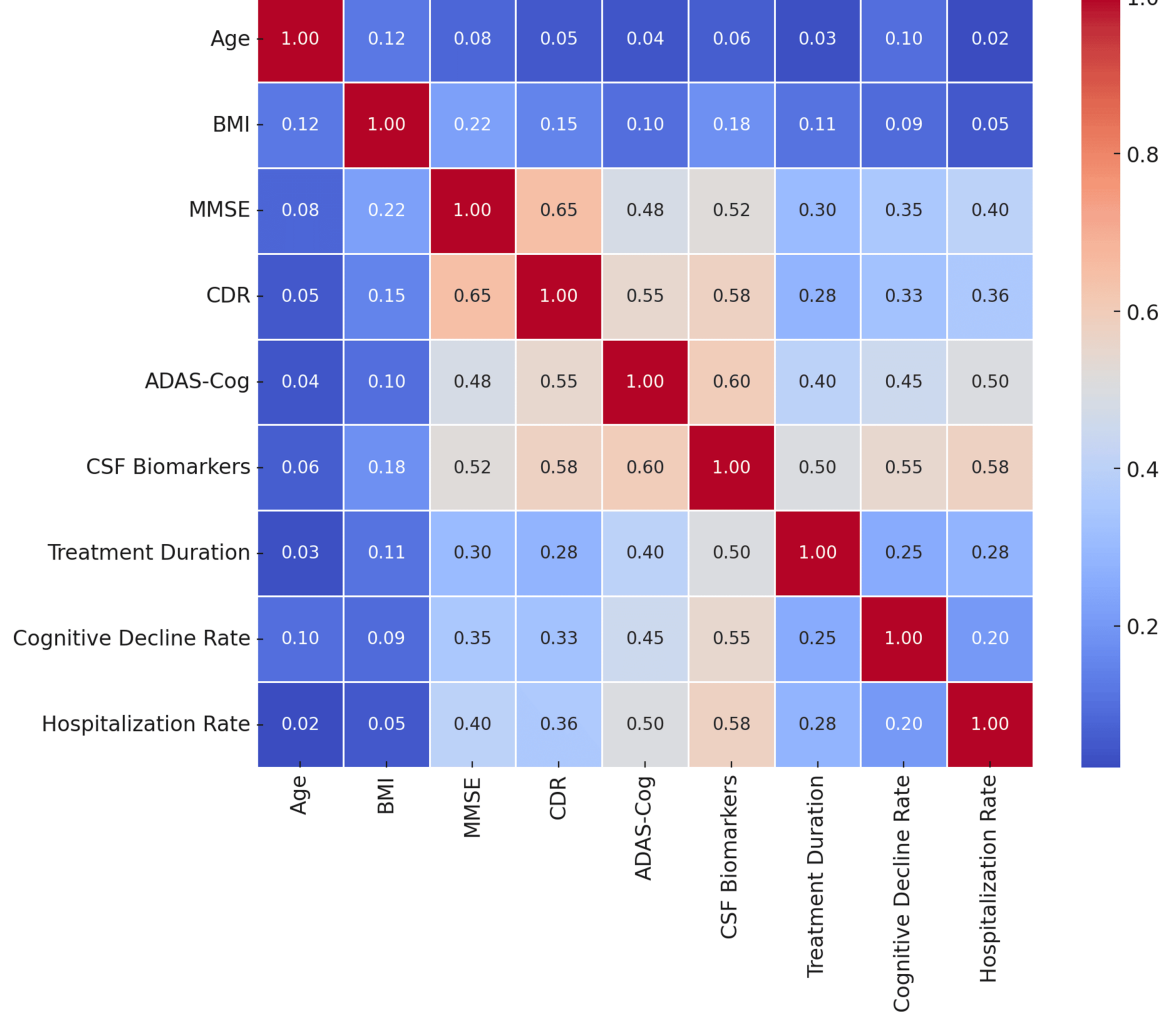
Heatmap of Correlations Between Key Variables MMSE, Mini-Mental State Examination; CDR, Clinical Dementia Rating; ADAS-Cog, Alzheimer's Disease Assessment Scale-Cognitive Subscale; CSF, Cerebrospinal Fluid; BMI, Body Mass Index

The Chi-square test was used to compare treatment groups on categorical variables. The baseline PET scan findings test was significant (χ²(1, N = 250) = 5.78, p = 0.016, φ = 0.15), demonstrating a notable distinction in amyloid positivity between groups. However, comparisons between the treatment groups based on APOE4 status (χ²(1, N = 250) = 0.29, p = 0.59, φ = 0.03), living status (χ²(3, N = 250) = 2.87, p = 0.41, Cramer’s V = 0.11), and adverse effects (χ²(1, N = 250) = 1.29, p = 0.25, φ = 0.07) showed no significant differences. Cohen's d and Hedges' g were applied as effect size estimates for key variables. The effect sizes for MMSE (d = 3.08, p < 0.001) and CSF biomarkers (d = 2.88, p < 0.001) were highly significant, suggesting large differences between groups in terms of cognitive function and disease pathology measures (Figure 5).

Key findings and interpretation

Concerning the cognitive outcomes from this study, rivastigmine and donepezil showed no significant differences, as all three percentages of improvement, stability, and decline were almost equal between the two groups. The mean cognitive decline rate was moderate, while equal hospitalization rates were observed between treatment arms. Nausea was reported in 21% of cases, dizziness in 18%, bradycardia in 8%, and gastrointestinal upset in 5%, with the majority classified as mild to moderate in severity. Yet, a majority of rivastigmine patients reported side effects, which may or may not have affected compliance with treatment. Neuroimaging studies show that most patients tested positive for amyloid, thus solidifying the diagnosis of AD. CSF biomarkers confirmed this, with higher tau levels correlating with neurodegeneration. The effect size calculations showed that MMSE scores and CSF biomarkers were strongly interrelated with cognitive outcomes, thus upholding the validity of these measures.

## Discussion

The present study offers a comparative evaluation of the effects of rivastigmine and donepezil in patients with AD and, as such, shows that both therapies provide equivalent cognitive benefits [6]. The primary outcome analysis demonstrated that, out of 100% of these patients, 33.2% showed improvement, 33.6% remained stable, and 33.2% declined, indicating that neither drug is appreciably more effective than the other in arresting cognitive impairment. The nearly identical percentages of patients who showed cognitive improvement, stability, or decline between the two groups suggest no superiority of either drug in altering the course of AD during the period considered. As one-third of both treatment groups improved, one-third remained stable, and one-third declined, these outcomes appear more randomized than therapeutically decisive. The absence of differences was also confirmed by statistical analysis of cognitive outcomes (χ²(2, N = 250) = 0.08, p = 0.96). Individual variability in the rate of disease progression, rather than drug effects, is likely the primary determinant of patient outcomes. Thus, the therapeutic choice between rivastigmine and donepezil should be individualized based on side effect profiles, comorbidities, and personal tolerance, rather than expectations of greater cognitive benefit from one over the other. This finding aligns with previous literature: ChEIs are beneficial in early to moderate AD stages but do not halt neurodegeneration in the long term [10]. While producing a similar cognitive outcome to donepezil, rivastigmine was associated with a higher incidence of side effects (52%), which likely affected patient adherence to treatment and their QOL. Adverse effects such as nausea, dizziness, and bradycardia may contribute to treatment discontinuation or noncompliance during the long course of AD; hence, drugs should be selected carefully based on individual patient tolerance [11].

This research encompasses several limiting factors that prevent differentiation between hospitalizations related to AD progression and those caused by drug-induced side effects. Hospitalization rates, therefore, cannot be attributed solely to disease severity or treatment tolerability. While the average hospitalization frequency of three visits was found to be comparable between the rivastigmine and donepezil groups, this measure alone cannot reliably evaluate the efficacy and safety of these treatments. Subsequent studies should try causality-based stratification of hospitalization in order to get a better picture of the clinical burden of various therapeutic agents. Distinguish between drug-induced adverse effects - e.g., bradycardia and gastrointestinal issues - versus disease-related complications, e.g., falls or secondary infections due to dementia progression. The study used hospitalization data and found an average of 1.48 hospitalizations (SD = 1.12) per patient. Hospitalization was recorded in nearly 75% of patients, indicating the considerable burden on healthcare services that is likely inevitable with AD and its complications [12]. While the two medications did not show statistically significant differences in hospitalization rates, comorbid conditions may have contributed to the observed burden. The study population showed high rates of chronic diseases: diabetes (52%), hypertension (51.2%), hyperlipidemia (51.2%), cardiovascular disease (49.2%), and stroke (53.2%) [13]. Vascular pathology has been shown to accelerate cognitive decline, suggesting that many patients may have had mixed dementia (with both AD and vascular contributions), which likely affected their response to ChEIs. Lifestyle factors such as smoking (34.8%) and frequent alcohol intake (34.4%) further emphasize the need for integrated lifestyle interventions as part of comprehensive dementia care [14].

The role of genetic and neuroimaging markers was also analyzed regarding AD progression; it was found that 46% of patients were identified as APOE4-positive. Previous research has found a similar link between this genetic variant and a higher risk for AD or cognitive decline. However, there were no significant differences in APOE4 status between treatment groups, which suggests that, in this study, genetic predisposition did not affect response to the drugs [15]. Neuroimaging results showed that 57.6% of patients were amyloid-positive on PET scans, confirming that the underlying Alzheimer's pathology was already present. A Chi-square test showed a significant p-value for amyloid positivity across groups (p = 0.016), indicating that some patients might have had higher amyloid levels at baseline, which may affect drug response [16]. CSF biomarker profiles were also closely associated with cognitive performance, with higher tau levels corresponding to more severe cognitive decline. These findings highlight how biomarkers could serve as potential predictors for overall disease progression and individual responses to treatment. This would support a frontline approach to personalized medicine in AD management [17].

In analyzing the data statistically, the t-test pointed to significant differences between treatment groups with respect to cognitive scores, treatment duration, and biomarker levels (p < 0.05). Age was not found to have a significant effect on rates of cognitive decline (p = 0.208) or hospitalization (p = 0.925), per one-way ANOVA. There appeared to be a near-significant effect on CSF amyloid levels (p = 0.058), which was not statistically significant. These analyses suggest that age, per se, does not appear to be a strong predictor of disease progression, whereas the biomarkers might serve as better predictors of cognitive decline [18]. This finding implies a personalized approach to treatment. Clinical relevance suggests that the choice between rivastigmine and donepezil should be made on a case-by-case basis, with consideration of side effect profiles, comorbidities, and genetic risk factors [19]. Rivastigmine is reported to have relatively more side effects affecting treatment compliance and patient QOL. More immediate considerations would include vascular-targeted therapies related to hypertension, smoking cessation, and lifestyle changes, against the background of a high prevalence of cardiovascular disease and stroke in this group, since possible cognitive benefits may accrue from these treatments along with any other drugs.

Future research should focus on long-term follow-up studies and RCTs comparing ChEIs across mixed patient populations. Biomarker-driven research using CSF amyloid and tau levels will further assist in predicting which patients will benefit most from specific drug interventions [20]. This study found a greater incidence of side effects among rivastigmine users, which is 52%. Meanwhile, there are strategies to manage and minimize these adverse effects: titration, slow-up titration schedules, or changing to alternative formulations (e.g., the transdermal rivastigmine patch), which alleviate gastrointestinal and cardiovascular side effects. Proactive monitoring of vital signs, educating patients about early detection of side effects, and supportive measures (e.g., antiemetics for nausea) can further increase tolerability. Hence, although the problem of side effects is worrisome, customized doses and formulation options can help in making therapy more adherent and efficacious in the clinical setting. Additional investigation into combination therapy or new mechanisms of action should refine treatment options for AD. Some of these findings, thus far, remain very encouraging, but some methodological issues must be acknowledged. Genetic differences, such as APOE status, which can affect treatment response, were not accounted for in this study. Also, the sample size was rather small, limiting the generalizability of the results. In addition, by the nature of its observational design, the study restricts causation interpretations, which necessitates RCTs to validate these results. Future studies should then focus on these limitations to enhance the applicability of this study in the clinical field.

## Conclusions

This retrospective cohort study has revealed that rivastigmine and donepezil have equal cognitive benefits in patients with AD, with no substantial difference found in rates of cognitive improvement, stabilization, or decline. On another note, rivastigmine generated a higher occurrence of side effects, which could potentially pose an issue in terms of treatment adherence and tolerability. These many comorbidities and hospitalizations further emphasize the complex healthcare needs of this patient population. Such observational design limitations restrict the possibilities for making causal inferences, and the findings may not be generalized due to the relatively small sample size from a single center. Furthermore, an inability to differentiate between hospitalizations due to disease progression and those following treatment side effects also limits interpretation. This study lacked adherence data, which would have greatly enhanced the evaluation of its real-world effectiveness. Thus, these results are best viewed as hypothesis-generating, laying a platform for proposed future, prospectively designed, biomarker-based investigations. Personalized treatment approaches for AD should address tolerability issues regarding side effects, as well as cancer treatments for comorbidities, genetic risk factors, and biomarker status, for the best possible outcomes. Future large-scale RCTs will need to confirm and expand upon these observations by stratifying patients based on the severity of the disease and their biomarker profiles.
